# ‘Low-Salt’ Bread as an Important Component of a Pragmatic Reduced-Salt Diet for Lowering Blood Pressure in Adults with Elevated Blood Pressure

**DOI:** 10.3390/nu11081725

**Published:** 2019-07-26

**Authors:** Kevin D. Cashman, Sorcha Kenny, Joseph P. Kerry, Fanny Leenhardt, Elke K. Arendt

**Affiliations:** 1 Cork Centre for Vitamin D and Nutrition Research, School of Food and Nutritional Sciences, University College Cork, T12 Y337 Cork, Ireland; 2 Muscle Foods Research Group, School of Food and Nutritional Sciences, University College Cork, T12 Y337 Cork, Ireland; 3 Cereals Research Group, School of Food and Nutritional Sciences, University College Cork, T12 Y337 Cork, Ireland

**Keywords:** sodium restriction, low-salt bread, hypertension, blood pressure regulation, crossover trial

## Abstract

Reformulation of bread in terms of salt content remains an important measure to help achieve a reduction in salt intake in the population and for the prevention of hypertension and elevated blood pressure (BP). Our fundamental studies on the reduction of salt on dough and bread characteristics showed that wheat breads produced with 0.3 g salt/100 g (“low-salt”) were found to be comparable quality to that produced with the typical level of salt (1.2%). This food-based intervention trial examined, using a 5 week cross-over design, the potential for inclusion of “low-salt” bread as part of a pragmatic reduced-salt diet on BP, markers of bone metabolism, and plasma lipids in 97 adults with slightly to moderately elevated BP. Assuming all sodium from dietary intake was excreted through the urine, the intake of salt decreased by 1.7 g/day, on average, during the reduced-salt dietary period. Systolic BP was significantly lower (by 3.3 mmHg on average; *p* < 0.0001) during the reduced-salt dietary period compared to the usual-salt dietary period, but there was no significant difference (*p* = 0.81) in diastolic BP. There were no significant differences (*p* > 0.12, in all cases) in any of the urinary- or serum-based biochemical indices of calcium or bone metabolism or in plasma lipids between the two periods. In conclusion, a modest reduction in dietary salt intake, in which the use of “low-salt” (i.e., 0.3 g/100g) bread played a key role along with dietary advice, and led to a significant, and clinically meaningful, decrease in systolic, but not diastolic, BP in adults with mildly to moderately elevated BP.

## 1. Introduction

Cardiovascular disease (CVD) is the leading global cause of mortality, accounting for just under a third of deaths [1,2]. Hypertension is one of the major controllable risk factors associated with CVD, especially heart attack and stroke [3,4]. This is of concern given that, based on 2008 data, the global prevalence of raised blood pressure (BP) amongst adults over 25 years is around 40% [2], and is estimated to cause 9.4 million deaths every year — more than half of the estimated 17 million annual deaths caused by total CVD [5]. With the estimate of CVD-related annual deaths projected to rise to 23.3 million by 2030, the burden of morbidity and mortality from hypertension and related cardiovascular diseases is currently one of the most urgent public health problems globally [6].

While a number of dietary factors play a significant role in the prevention of hypertension and the maintenance of normal BP [7], salt reduction has been identified as one of the most cost-effective interventions for reducing the burden of CVD with the potential for saving millions of lives each year [8]. The World Health Organization’s (WHO) ‘Global Action Plan for the Prevention and Control of Non-Communicable Disease’ include a voluntary global target of a 30% relative reduction in mean population intake of salt/sodium by 2025 [9]. The WHO’s prioritization of salt reduction in the early 2000s led to an increase in the number of countries implementing salt reduction strategies, many of which were government-led with population salt targets [10]. For example, in Ireland a programme was commenced in 2003 [11] with an initial goal to achieve voluntary, gradual, sustained, and universal reductions in the salt content of processed and prepared foods. At that time, bread and cured/processed meats collectively contributed 45% of total salt intake of Irish adults [12]. The programme led to the average level of salt in white bread falling from 1.4 g/100g in 2003 to 1.1/100g in 2011 (a 21% reduction), with even greater reductions in some processed/prepared food types, and less, or none, in others [11]. Data from two consecutive national nutrition surveys of adults in Ireland showed that during 2009–2010 (the most recent survey), bread and cured/processed meats collectively contributed 0.95 g less to daily salt intake compared to that during 1997–1999, but they still contributed 40% of total salt intake [13]. 

While viewing the actions that many countries were taking to reduce salt consumption in their populations as promising, in 2013, the WHO emphasised that further research focusing on salt reduction strategies remained paramount [9]. Reformulation of bread in terms of salt content remains an important measure to help achieve a reduction in salt intake in the population. Our fundamental studies on the reduction of salt on dough and bread characteristics showed that wheat breads produced with 0.3 and 0.6 g salt/100 g (which can be labelled as “low-salt” and “reduced-salt”, respectively [14]) were found to be comparable to that produced with the typical level of salt (1.2 g/100 g) in terms of dough rheology, baking quality characteristics, and sensory attributes [15]. Modelling of data from the national nutrition survey of Irish adults (*n* = 1379) showed that switching from regular bread to a low-salt equivalent would reduce the average salt intake by 0.8 g/day, and even more if non-consumers within the survey data were excluded (unpublished data). 

While there is little disagreement that decreased salt-intake lowers BP [4], especially in those with elevated BP [16], and may have additional benefits in terms of bone health by lowering urinary calcium loss [17,18], there has been some concern that it might also lead to possible adverse effects in health. For example, decreased sodium intake results in reduced blood volume, which without a concurrent reduction in blood lipids can lead to an increased concentration of lipids in the blood [4].

Therefore, the aim of this food-based intervention trial, was to examine, using a 5-week cross-over design, the potential for inclusion of low-salt bread as one component of a pragmatic reduced-salt diet on BP, markers of bone metabolism, and plasma lipids in adults with slightly to moderately elevated BP.

## 2. Materials and Methods

### 2.1. Subjects

Ninety-seven apparently healthy adults (mean age 46.7 years) with slightly to moderately elevated BP (pre-hypertension to stage 1 hypertension: minimum >120/80 mmHg) were recruited from the free-living community in the greater Cork area, Republic of Ireland, via advertisements placed around the University as well as across the location. The mean age, height, weight, body mass index (BMI), systolic and diastolic BP, daily urinary Na output, as well as number of current smokers and ratio of males to females are provided in Table 1. Inclusion criteria were: consenting adult Caucasian men and women, aged ≤ 65 years with a seated office systolic BP ≥ 120 and < 160 mmHg and/or a diastolic BP ≥ 80 and < 95 mmHg (based on the mean BP across 3 screening visits as part of a pre-intervention phase of the trial; see Section 2.3), and willingness to consume study breads. Volunteers were excluded if they were taking anti-hypertensive medication or any medications known to interfere with BP or calcium or bone metabolism. Severe medical illness: celiac disease, hypercalcemia, known intestinal malabsorption syndrome, excessive alcohol use (>14 drinks/week), and pregnancy were also reasons for exclusion. 

### 2.2. Ethical Considerations

The study protocol was approved by the Clinical Research Ethics Committee of the Cork Teaching Hospitals, University College Cork (UCC), Ireland (Ref ECM 5 (9) 05/12/06) and was conducted in accordance with the principles of Good Clinical Practice and the Declaration of Helsinki. Detailed information about the study was provided to all volunteers, and all eligible participants provided their written consent. The trial was registered at clinicaltrials.gov: Identifier NCT04003597.

### 2.3. Design and Conduct of Study

The study consisted of a randomized crossover trial of the effect of reduced-salt intake or usual-salt intake for 5 weeks on BP (primary outcome) and biochemical markers of calcium and bone metabolism as well as plasma lipids (secondary outcomes) in adults with slightly to moderately elevated BP. 

Potential volunteers were screened for BP over 3 weeks preceding the intervention phase of the trial during which seated office systolic and diastolic BP were measured (see Section 2.5) weekly, and those with systolic BP ≥120 and <160 mmHg or a diastolic BP ≥80 and <95 mmHg, were considered eligible.

The dietary intervention phase of the trial was designed in two successive dietary periods, each of 5 weeks. Subjects were randomly assigned to the reduced-salt diet or their usual-salt diet (control) for 5 weeks, followed by crossover to the alternative dietary regimen for a further 5 weeks. At baseline (week 0), the subjects visited the Human Nutrition Studies Unit within UCC in a fasting state and had their BP measured. Prior to the baseline visit, subjects received instructions on how to collect a 24-h urine sample and were provided with a suitable container(s) for collection. In brief, participants were informed to keep their storage container in a cool dark place, a refrigerator if possible, throughout the 24-h collection period until they bring it back to the assigned drop area within the University centre. They were informed they may require a receptacle/vessel to collect urine into and then add to the container. On the day of the collection, they were asked to start the 24-h urine test by urinating directly into the toilet. They were told not to save this urine. The 24-h collection began after this passing, and they were asked to write the date and time on their container. They were instructed that for the next 24 h, they needed to collect all urine in the container. The 24-h period ends when the participant wakes the next day and passes their first urine of the day. They were instructed to again write the date and time on their container. On the day of the baseline visit, subjects brought the 24-h urine sample collected from the previous day and deposited it in a cold room area dedicated for such collections. A blood sample was taken from each subject between 08.30 am and 10.30 am by a trained phlebotomist and anthropometric measures, including height and weight, were taken, as described previously [19]. A health and lifestyle questionnaire, which assessed physical activity, general health, smoking status, and alcohol consumption was completed by each subject. Subjects randomized to start on the reduced-salt diet were asked to restrict their consumption of dietary salt using a combination of pragmatic dietary advice as well as the replacement of bread and a limited number of other foods with equivalent foods which had lower salt content, these were provided to the participants. At the beginning of the salt restriction period, a research nutritionist provided the subjects with a list of the common salt-containing foods (salted and naturally salty) and they were asked to limit the consumption of such, as feasible. The subjects received in-house prepared ‘low-salt (0.3 g/100 g)’ brown or white sliced pan bread (see Section 2.4) as well as no-salt margarine (no salt Flora, Unilever, UK), and were given luncheon meats with no added salt (cooked turkey and beef; see below), if desired (optional). Subjects commencing the trial on the control diet were allowed to follow their usual diet but were asked to consume an in-house produced brown or white sliced pan bread equivalent in composition to the low-salt version but with its more typical salt content (1.2 g/100 g). For both dietary periods, subjects were asked to maintain their usual pattern of bread consumption but to replace their usual bread with the study breads. The subjects were not given target portion sizes of bread to achieve within the study period, just to maintain their usual intakes. Regular-salt and reduced-salt chilled ready meals were also made available to participants, but volunteers did not avail of the offer mainly due to the logistical issues of transfer to home and their storage in a freezer. Finally, as restriction of salt intake to <5g/day has been shown to reduce potassium intake in free-living subjects with mildly elevated blood pressure [20], volunteers in the present study were offered Knorr Vie shots, which deliver fruit and vegetables in a single convenient 100 ml drink, as a means of augmenting their intake of potassium-rich fruit and fruit and vegetables during the reduced-salt dietary period. However, only very few volunteers availed of this offer. At the crossover phase, subjects switched to the alternate diets for a further 5 weeks. 

During the intervention phase, each participant made 2 further visits to the study unit, one each on the last day of dietary period 1 (week 5) and dietary period 2 (week 10). At these visits, BP measurements were taken as well as other anthropometric measures, including height and weight. An overnight fasting blood sample was also taken from each participant between 08:30 a.m. and 10:30 a.m. by a trained phlebotomist, and on both visits, the subjects provided a 24-h urine sample collected the previous day. Following blood sampling the participants received their breakfast within UCC.

Participants met the research staff weekly to receive breads as well as no-salt margarine and luncheon meats (where applicable), and at these meetings the staff promoted compliance with the intervention and encouraged completion of the study protocol. The trial ran from January 2008 to July 2010.

### 2.4. Production of Low-Salt and Usual-Salt Bread and Luncheon Meats with No Added Salt

‘Low-salt’ white and brown pan breads (0.3 g salt/100 g; a level shown to be the minimum required to provide the functional properties required in bread-making [15]) and equivalent breads with usual-salt levels (1.2 g salt/100 g) were manufactured in the experimental bakery at the School of Food and Nutritional Sciences, UCC. In brief, the breads were produced using the following raw materials: commercial bakers’ wheat flour (containing 12 g protein and 12 g water/100 g; Odlums Ireland), lyophilised yeast (Puratos, Belgium), deionized water (water level set to 63%) and salt (either 0.3 or 1.2 g/100 g), and the conditions of preparation (mixing, proofing, and baking) followed that as described in detail elsewhere [15,21]. There were no taste enhancers included in the low-salt bread in lieu of the salt. Considering the central role of these breads in the diets to be adhered to for 5 weeks each, the consumer acceptability of the breads was tested and demonstrated in advance of the present study using a trained panel of 8 assessors to undertake descriptive sensory analysis, described in detail elsewhere [15].

The commercial availability of low-salt luncheon meats was very limited at the time of the study and thus luncheon meats with no added salt were produced in-house within the meat processing facility at the School of Food and Nutritional Sciences, UCC. In brief, beef silverside (aged for 21 days at 4 °C) and Turkey crown (supplied 3 days post-slaughter) were placed in stainless steel meat-molds containing polyethylene liners. The meat-molds shaped the meats to assist with the subsequent slicing process. All meats were cooked in a Ness oven (Ness and Co., GmbH, Germany) at an oven temperature of 85 °C and relative humidity of 90% until they reached a product core temperature of 74 °C. The cooked meats were cooled with the aid of a cold-water shower until the product core temperature reached 30 °C, and transferred to a cold room at 2 °C, resulting in the product core temperature being reduced to less than 5 °C in a total cooling time of 7 h. These were supplied in daily portions with instructions to keep frozen until the day of use.

### 2.5. Blood Pressure Measurements

BP (systolic and diastolic) was measured in accordance with the European Society of Hypertension guidelines [22]. Office BP was measured on fasted study participants, between 8:30 a.m. and 10:30 a.m., prior to blood sampling, on the same arm at each visit and in accordance with a standardized protocol. BP was measured in a seated position after the study participant sat quietly for 10 min in a quiet and warm room. Office BP was measured three times in succession using a validated Omron HEM-705-IT fully automatic digital blood-pressure monitor (Omron Healthcare Europe B.V., Hoofddorp, The Netherlands) with 2 min intervals between readings and the average of the last two measurements was recorded.

### 2.6. Preparation of Biological Samples

The volumes of 24-h urine collections were recorded, and portions of urine were acidified using 0.36 M HCl and stored at −20 °C from the morning of collection until required for analysis. Overnight fasting blood samples were collected by venepuncture into vacutainer tubes containing either no additive or K_3_EDTA and processed to serum and plasma, respectively. The samples were immediately stored at −70 °C until required.

### 2.7. Biochemical Analysis

#### 2.7.1. Urinary Creatinine

Creatinine was determined in 24-h urine samples by a colorimetric procedure using a diagnostic kit (Metra Creatinine Assay Kit, Catalogue No. 8009, Quidel Corporation, CA, USA). The intra-assay CV was 1.6%. Urinary creatinine was used to identify suspected inaccurate urine collections (i.e., urinary creatinine <4 mmol/day for women, or <6 mmol/day for men [23]), which were them excluded from the statistical analyses.

#### 2.7.2. Urinary Calcium, Sodium, and Potassium

Calcium (Ca) was analysed in duplicate in 24-h urine samples by atomic absorption spectrophotometry (Spectr AA-600; Varian Australia Ltd., Victoria, Australia) after appropriate dilution with LaCl_3_ solution (5 g/L; BDH Ltd., Poole, Dorset, UK). A range of Ca standards was used to obtain a Ca calibration curve. The intra-assay CV for Ca was 2.8 %. Sodium and potassium were determined in duplicate in 24-h urine samples by flame photometry (Jenway PFP7; AGB Ltd., Dublin, Ireland) using appropriate sodium and potassium standards. The intra-assay CV for sodium and potassium was 3.8% and 4.5%, respectively. The accuracy of the mineral analysis was assured in each analytical run by appropriate recovery of mineral in samples of a reference urine (Seronorm Trace Elements™: Urine, Nycomed, Zurich, Switzerland).

#### 2.7.3. Urinary N-Telopeptides of Type I Collagen

The concentration of N-telopeptides of Type I collagen (NTx) was measured in 24-h urine samples by an enzyme-linked immunosorbent assay (ELISA) (Osteomark^®^, Unipath Ltd., Bedford, UK). The intra-assay CV was 5%. Urinary NTx concentrations were expressed relative to urinary creatinine concentrations.

#### 2.7.4. Serum C-Telopeptide of Type I Collagen

The concentration of C-telopeptides of Type I collagen (CTx) was measured in serum by an ELISA (Serum CrossLaps^®^ ELISA, Nordic Bioscience Diagnostics, Herlev, Denmark). The intra-assay CV was 4.2%.

#### 2.7.5. Serum Osteocalcin and Bone-Specific Alkaline Phosphatase

The concentration of osteocalcin was measured in serum samples using an ELISA (Metra™ Osteocalcin EIA Kit, Quidel Corporation, CA, USA). The intra-assay CV was 6.0%. Bone-specific alkaline phosphatase (*EC* 3.1.3.1) activity was measured in serum samples using an ELISA (Metra™ BAP EIA Kit, Quidel Corporation, CA, USA). The intra-assay CV was 5.0%.

#### 2.7.6. Serum 25-Hydroxyvitamin D

The concentration of 25-hydroxyvitamin D (25(OH)D) was measured in serum samples using an ELISA (OCTEIA^®^ 25-Hydroxy Vitamin D, Immuno Diagnostic Systems, Ltd., Boldon, UK). The intra-assay CV for the ELISA method was 5.9%. This ELISA assay is used for the quantitative determination of 25(OH)D, further details of which have been described previously [24]. The quality and accuracy of serum 25(OH)D analysis in our laboratory was assessed and assured on an ongoing basis by participation in the Vitamin D External Quality Assessment Scheme (DEQAS, Charing Cross Hospital, London, UK).

#### 2.7.7. Serum Intact Parathyroid Hormone

The concentration of intact parathyroid hormone (PTH) concentrations was measured in serum using an ELISA (Intact parathyroid hormone, MD Biosciences Inc., St. Paul, MN 55108). The intra-assay CV was 3.4%. 

#### 2.7.8. Plasma Lipids

Total cholesterol, high-density lipoprotein (HDL) cholesterol and triacylglycerol (TAG) concentrations were analysed in plasma samples using enzymatic colorimetric assays (Randox laboratories Ltd., UK), whereas low-density lipoprotein (LDL) cholesterol was calculated using the Friedewald formula [25]. The intra-assay CVs for TAG, total- and HDL-cholesterol were 7.7%, 6.5%, and 2.5%, respectively. TAG values were corrected for free glycerol by subtracting 0.11 mmol/L from the value obtained.

Inter-assay variation in urinary minerals, biochemical markers of bone turnover, and serum 25(OH)D and PTH as well as plasma lipids was avoided by analysing all samples from an individual in the same run. 

### 2.8. Sample Size Calculation

Sample size for the cross-over intervention trial was calculated based on systolic BP data. We used an estimated standard deviation of paired response differences of 0.5 mmHg based on the results of a previous unpublished study of modest salt restriction in Irish adults. Modelling of data from consumers amongst the national nutrition survey of Irish adults showed that switching from regular bread and regular cured/processed meats to low-salt and no added salt equivalents respectively, would reduce the average salt intake by 1.5 g/day. Linear regression of data from a meta-regression analysis of 17 trials of modest reductions in salt intake predicts that for every 1 g decrease in salt intake, systolic BP in normotensive and hypertensive subject will decrease on average by 0.60 and 1.18 mmHg, respectively [26]. While our subjects by design would have elevated BP, we decided to take a more pessimistic estimate by using the average systolic BP reduction value for the normotensive and hypertensive subjects (0.9 mmHg) and thus, our predicted decrease in systolic BP arising from a reduction in salt intake within our study was 1.4 mmHg. In total, 95 participants were required to complete the study which would give it 80% power to detect a mean difference in systolic blood pressure during the two crossover phases of the study of 1.4 mmHg at the 5% significance level.

### 2.9. Statistical Analysis

Statistical analysis of the data was conducted using SPSS^©^ for Windows^TM^ Version 21 (SPSS, Inc., Chicago, IL, USA). The distributions of all variables were tested with Kolmogorov–Smirnov tests and any non-normally distributed variables were log-transformed which improved their normality and allowed for parametric tests of significance for all variables. Descriptive statistics (mean and SD) were determined for all variables.

Differences between the two dietary regimens in BP, urinary minerals, biochemical indices of calcium and bone metabolism, and plasma lipids were analysed by the appropriate analysis for a crossover trial with continuous data as described by Jones and Kenward [27]. Two-sample *t* tests and ANOVA were used to test hypotheses about direct treatments effects (i.e., usual versus reduced salt intake), carry-over effects and interactions. A carry-over (or residual) effect is defined as the effect of the treatment from the previous time period (i.e., dietary period 1) on the response at the current time period (i.e., dietary period 2). It occurs when the effect of a treatment given in the first time period persists into the second period and distorts the effect of the second treatment. As such, a carry-over effect, if evident, may bias the direct treatment effects. In a simple two period, two treatment (sometimes called a AB/BA) cross-over trial, such as the present study, the treatment effect can be tested by estimating the mean difference, whereas for the carry-over effect (the effect of period), the first period mean minus second period mean can be estimated for each treatment and the null hypothesis that the difference between these two mean differences is zero can be tested. Regression models were also performed to account for potential confounders for these outcome variables. As a secondary analysis, we also stratified participants by baseline systolic BP < 140 or ≥ 140 mmHg and repeated the carry-over and treatment effect analysis of systolic and diastolic BP within both groups separately. The difference in response between these two subgroups was tested using an unpaired Student’s *t*-test.

## 3. Results

**Figure 1:**
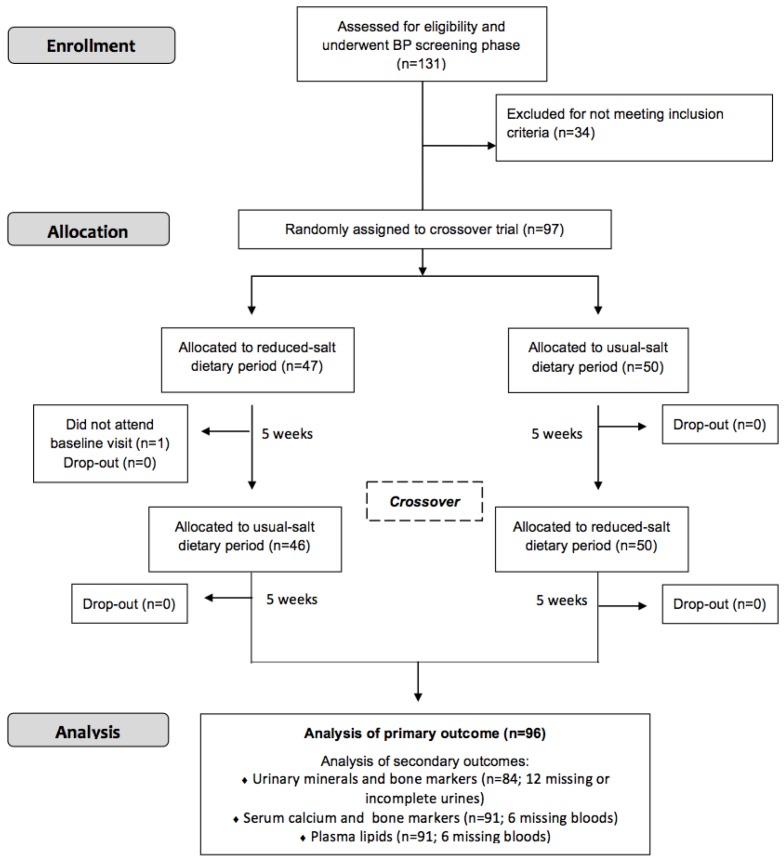
CONSORT flow diagram of participants.

Using the systolic BP measurements at baseline, about 56%, 41%, and 3% of participants had readings between 120–139, 140–159, and 160 + mmHg, respectively. Of the 97 participants who were randomly assigned, 96 finished the trial (Figure 1). There was no significant difference for mean body weight between the two dietary periods (mean ± SD: 80.9 ± 14.5 versus 80.6 ± 14.3 kg; *p* = 0.48).

The effects of the reduced-salt diet for 5 weeks on BP, urinary minerals, and urinary- and serum-based biochemical markers of calcium and bone metabolism are shown in Table 2. Statistical analyses showed that there were no significant carry-over effects evident for systolic or diastolic BP (*p* > 0.47 for both) or for any of the biochemical indices (*p* > 0.13, in all cases). In terms of treatment effects, the mean daily urinary sodium output during the reduced-salt dietary period was significantly lower (*p* < 0.0001) than that during the usual dietary period, and assuming all sodium from dietary intake was excreted through the urine, the intake of salt decreased by 1.7 g/day, on average, during the reduced-salt dietary period. Systolic BP was significantly lower (by 3.3 mmHg on average; *p* < 0.0001) during the reduced-salt dietary period compared to the usual-salt dietary period, but there was no significant difference (*p* = 0.81) in diastolic BP of the adults between the two periods.

In the secondary analysis, where participants we stratified by baseline systolic BP < 140 or ≥ 140 mmHg, there was again no significant carry-over effects evident for systolic or diastolic BP (*p* > 0.22 in all cases). There was no significant difference in diastolic BP between the two periods in either the group with baseline systolic BP < 140 or ≥ 140 mmHg (*p* > 0.38, in both cases). Systolic BP was significantly lower (*p* < 0.008, in both cases) during the reduced-salt dietary period compared to the usual-salt dietary period in both subgroups; by 2.9 ± 7.9 (*n* = 54) and 4.2 ± 8.3 (*n* = 42) mmHg on average, in the baseline systolic BP < 140 or ≥ 140 mmHg groups respectively, with no significant difference between the two (*p* = 0.43).

There were no significant differences (*p* > 0.12, in all cases) between the two dietary periods for mean urinary potassium or Ca, or for mean concentration of serum 25(OH)D or PTH (Table 2). Likewise, there were no significant differences (*p* > 0.14, in all cases) between the two dietary periods for mean serum osteocalcin, bone-specific alkaline phosphatase or CTx concentration, or for mean urinary NTx concentration (Table 2). 

The effect of the reduced-salt diet for 5 weeks on plasma lipids are shown in Table 3. Statistical analyses showed that there were no significant carry-over effects evident for any of the plasma lipid indices (*p* > 0.67, in all cases). There were no significant differences (*p* > 0.18, in all cases) between the two dietary periods for mean plasma total-, HDL- or LDL-cholesterol concentrations or mean plasma TAG concentrations.

The significant difference in systolic BP (*p* < 0.0001 to *p* = 0.001, in all cases) remained in various regression models which included potential confounders (age, sex, smoking status, baseline parameter under investigation, and then weight at baseline or weight change, and baseline urinary sodium) (See Table S1 in Supplementary material). The lack of significant differences (*p* > 0.1, in all cases) in diastolic BP as well as biochemical markers of bone metabolism or plasma lipids remained in various regression models which included these potential confounders. 

## 4. Discussion

It is well-accepted that lifestyle modifications that achieve even small reductions in BP are likely to reduce rates of CVD significantly [28]. The present study showed that a modest reduction in dietary salt intake, in which the use of low-salt bread played a key role in addition to alterations stemming from the pragmatic dietary advice, led to a significant, and clinically meaningful, decrease in systolic, but not diastolic, BP in adults with pre-hypertension to stage 1 hypertension. There was no effect of the reduced salt intake on biochemical markers of bone metabolism or on plasma lipids. While the study by design was in adults with existing elevated BP, this is regrettably a common occurrence within Ireland and elsewhere, with about 4 in 10 adults aged over 25 years having systolic BP > 115 mmHg [2], above which the relative risk for ischemic heart disease, stroke, and other CVDs begins to rise [29]. There is a graded relationship between BP and CVD, and strokes occur in a large number of people whose BP levels are in the ‘high-normal’ range, outside the current classification criteria for hypertension [30]. From a public health impact perspective, if generalized to the population, a 3 mmHg reduction in systolic BP would reduce the incidence of stroke (up to ~14%) and of ischemic heart disease (up to ~11%) [31]. By extrapolation, that could mean a reduction in stroke deaths of ~350 per year in Ireland alone (with a similar reduction in the number of non-fatal disabling strokes), and a reduction in deaths due to ischemic heart disease of 642 per year [32]. There was no corresponding lowering effect of the reduced salt intake on diastolic BP in the present study, however, reductions in diastolic BP with salt restriction are much smaller than that observed for systolic BP [16] and the study was not powered for diastolic BP. Only 29% of participants in the present study had baseline diastolic BP ≥ 90 mmHg compared to 43% with baseline systolic BP ≥ 140 mmHg, as the BP cut-offs for stage 1 hypertension.

The magnitude of the systolic BP-lowering effect of reduced salt intake (i.e., −1.94 mmHg/g salt) in the present study of adults with elevated BP was greater than that reported from a linear regression as part of a meta-regression analysis of 17 trials of modest reductions in salt intake amongst hypertensive subjects (−1.18 mmHg/g salt) [26], but aligns closely with that reported from the landmark Dietary Approaches to Stop Hypertension (DASH)-Sodium trial by Sacks et al. [16]. In that trial, participants with pre-hypertension or hypertension stage 1 were assigned to either a control diet or the DASH diet (rich in vegetables, fruits and low-fat dairy products, and has been shown to be able to lower BP of its own right [33]) and with high, intermediate, and low levels of sodium for 30 consecutive days [16]. The trial clearly showed that within the control diet, reducing the sodium intake from the high (equivalent to 8.25 g salt/day) to the intermediate level (equivalent to ~6.2 g salt/day) significantly reduced systolic BP by 2.1 mmHg, and reducing the sodium intake further from this intermediate level to a low level (equivalent to ~3.7 g salt/day) led to an additional significant reduction of 4.6 mm Hg [16]. This would translate into a −1.02 and −1.84 mmHg/g salt BP-lowering response, respectively. Mean salt intake in the present study (based on daily urinary sodium output) decreased from 6.3 g/day to 4.5 g/day. The effects of sodium within the DASH-Sodium trial were greater in participants with hypertension than in those without hypertension [16]. In the present study, those with baseline systolic BP ≥ 140 mmHg had a greater reduction in systolic BP compared to those with baseline systolic BP < 140 mmHg, although the difference was not statistically significant possibly due to a lack of power with subject numbers in the two sub-groups. Within the DASH-Sodium trial, the reduction in salt intake was achieved by daily menu planning and substituting regular salted bread, peanut butter, and spaghetti sauce, amongst others, with low-salt varieties of these foods [34]. It should be noted that standardized recipes and cooking procedures were followed, and all food was provided within the trial. The feasibility and effectiveness of such dietary interventions within free-living individuals has been questioned [35].

Another food-based intervention study compared the effect on BP of a low-sodium (equivalent to ~5.5 g salt/day) plus high-potassium (K) diet or a moderate-sodium (equivalent to ~10 g salt/day) DASH-type diet for 4 weeks compared to a control diet (equivalent to ~12.8 g salt/day) within community-dwelling subjects with elevated BP [35]. The subjects, after dietary advice, selected and prepared their own foods, and the major dietary changes required to achieve the reduction in sodium intake were use of salt-free bread and the avoidance of high-sodium foods. Compared to the control diet, the low-sodium plus high-K diet led to significant reductions in systolic and diastolic BP of 3.5 and 1.9 mmHg respectively, whereas the moderate-sodium DASH-type diet led to 1.8 mmHg significant reduction in systolic BP [35]. In a second community-based intervention study by the same group, the impact of a self-selected low-sodium (equivalent to ~5.5 g salt/day) plus high-K diet versus high-sodium (equivalent to > 13 g salt/day) for 4 weeks on BP of adults (92 normotensive and 16 hypertensive) was examined [28]. The low-sodium, high-K significantly reduced systolic BP by 2.5 mmHg compared to the high-sodium period, which was achieved by sodium supplements. Sodium reduction was again achieved by use of salt-free bread, salt-free margarine, salt-reduced stock powder and sodium-free baking powder, as well as advice on avoidance of high-sodium foods [28]. In both studies, however, there were changes in nutrient intake (particularly K) other than just sodium and decreased-sodium in the context of increased K appears to exert a greater BP-lowering effect that sodium restriction alone [35,36]. There was no change in K intake (as reflected by daily urinary output) in the present study. Salt-free bread, as used in the two studies mentioned above [28,35], while clearly of benefit in terms of its contribution towards lowering overall salt intake, has technological challenges in terms of its production. For example, our fundamental studies on the stepwise reduction of salt in wheat breads from 1.2 to 0 g/100 g showed that omission of salt entirely led to a significant reduction in dough and bread quality, and a less appealing taste profile [15], as well as a reduction in shelf-life [21] and level of staling after five days of storage [15].

This is the first study, to our knowledge, which has tested ‘low-salt’ bread, as fitting with the food regulatory definition, as a core element of the wider pragmatic dietary strategy for reducing salt intake, and consequently BP. An older study which assessed the impact of salt restriction on BP of a group of adults with mildly elevated BP used ‘a low-sodium bread’ together with advice on choosing low-sodium alternate foods [37]. The study showed that a 7.1 and 4.2 mmHg reduction in systolic and diastolic BP respectively, was achieved during the 24-week salt restriction dietary period in which mean salt intake dropped from 8.2 to 4.1 g/day [37]. The low-sodium bread, however, still contained 0.5 g sodium/100 g bread [37], although it has also been reported as ‘low-salt bread (0.5%)’ in another publication [20]. Either way, these would not fit with the low-sodium/salt definition of the European Commission (at ≤0.12 g sodium/100 g or ≤0.3 g salt/100 g) [14], or the low-sodium/salt definition of US Food and Drug Administration (≤0.14 g sodium/reference amount customarily consumed (50 g for bread)) [38]. As mentioned earlier, we have shown that bread with 0.3 g salt/100 g was comparable to that produced with the typical level of salt (1.2 g/100 g) in terms of dough rheology and bread-baking performances of wheat dough [15]. A sensory evaluation of bread regarding the influence of low-salt on changes of flavour during the staling process showed no significant differences between breads with salt levels of 0.3, 0.6 and 1.2 g/100 g [15]. An older study of Irish consumer preferences showed that while bread with the highest available salt level (1.2 g/100 g) was most preferred, those with 0.4 g/100 g salt still had acceptable sensory properties [39]. However, we have previously shown that bread made with 0.3 g salt/100 g had a reduction in shelf-life by two days compared to use of 1.2 g salt/100 g within bread [21]. This is not surprising as salt acts as a preservative in bread, as well as its roles in imparting flavour, improvement of texture and control of yeast growth and fermentation rate [40]. Of note, however, we have also shown that addition of sourdough with its antifungal activity, or the more traditionally-used calcium propionate, prolonged the shelf-life of low-salt breads by 12–14 and 10–12 days, respectively [21]. Selected sourdough strains are also capable of improving flavour in salt-reduced bread [40]. 

Data from a number of nationally representative nutrition surveys in Ireland over the period 2003–2011 highlight how bread alone contributes 22%, 20%, 21%, and 12% to the mean daily intake of salt in adults (18–64 years), teenagers (13–17 years), children (5–12 years), and young children (1–4 years), respectively [13]. Moreover, despite a 21% reduction in average levels of salt within bread in Ireland over the period 2003 to 2011 [11], a recent monitoring report of salt content in processed foods showed that average levels of salt in white or brown bread have not decreased in the period from 2011 to 2018 [41]. The average salt content of white and brown bread sampled in Ireland in 2018 was 1.2 g/100 g [41], similar to that of the regular bread used in the present study. Within the same nutrition surveys, cured/processed meats were shown to contribute between 16–20% to the mean daily intake of salt, dependent on age-group [13]. Using cooked ham as one example of cured/processed meats in Ireland, despite a 15% reduction in average salt levels over the period 2004 to 2015 [41], the average salt content in 2015 was 2.1 g/100 g. These data highlight the continuing need to consider further reductions of salt content of bread and cured/processed meats as important potential contributors to a dietary strategy for lowering population salt intake in children and adults, with associated benefits in relation to maintenance of normal BP. 

There was a lack of effect of the reduced salt intake on markers of bone metabolism in the present study. In general, intervention studies in which the salt intake was modified in premenopausal women have reported a lack of effect on markers of bone metabolism, and the effect in studies of postmenopausal women is mixed with some showing an increase in markers of bone resorption and others no effect (reviewed in [42]). In addition, the differential in sodium intake in these studies of postmenopausal women (range: 110–250 mmol/day) [42] far exceeds that in the present study (28 mmol/day). The lack of effect of the reduced salt intake on plasma lipid in the present study is in agreement with that of other modest salt reduction trials [35,43], although it has been suggested that it may take a longer period of dietary intervention to detect an effect [35]. 

While a major strength of the present study was its cross-over intervention design with sufficient power to detect a 1.4 mmHg difference in systolic BP, a number of limitations of the present work should also be noted. To reduce the burden on the participants, single 24-h urine collections were used at baseline and at the end of each dietary period. The International Consortium for Quality Research on Dietary Sodium/Salt (TRUE) has again recently highlighted that to accurately estimate usual dietary sodium at the individual level, at least 3 non-consecutive complete 24-h urine collections obtained over a series of days are needed [44]. Using the 24-h urine collection, the estimated mean salt intake of the present group of adults at baseline (at ~7g/day) was lower than that within the general adult population, aged 18–64 years, (mean, 9.7 g salt/day; based on analysis of first void urine samples within the national nutrition survey [13]). This may be due to the single 24-h urine collection procedure, or possibly due to the fact the subjects with elevated BP were aware of the role of salt and had already made dietary restrictions. These same reasons may also explain the relatively low salt intake (based on urinary sodium) by the participants during the usual salt dietary period. Nevertheless, the relative change in urinary sodium output between dietary periods, as measured by single 24-h urine collections in the present study, provides some indication of the change achieved in average salt intake by the group. As there can be large day-to-day variation in salt intake [45], the 24-h urine collection used in the present study also limited its applicability as a dietary adherence measure to allow for a compliance-based analysis of the effect of the reduced-salt intake on BP, as an additional data-analysis approach. It should be stated, however, that the intention to treat analysis, as used in the present study, is considered the gold standard as it allows the investigator to draw accurate (unbiased) conclusions regarding the effectiveness of an intervention [46]. In some studies, subjects were required to select and prepare all their own foods, including bread, following dietary advice on salt restriction, and thus the level of dietary compliance in such studies may be representative of dietary changes that are achievable in a group of motivated individuals living in the community. In the present study, the low-salt bread, in particular, was provided to the participants as a core part of the reduced salt dietary strategy. We felt this was important and warranted in light of levels of salt added to commercial breads and the relative lack of commercial availability of low-salt bread within Ireland. The present study did not record the consumption of the study foods each day of the intervention periods as we felt it would be too onerous, and this is a limitation, however, we had not set a specific target portion size of bread to be achieved but rather to follow usual dietary consumption of bread intake but with the study breads. Finally, it should also be stressed that there will be some patients for whom adequate control of blood pressure cannot be achieved by a reduction in salt intake alone and these will need to adhere to the guidance provided by their clinical care provider.

## 5. Conclusions

The present study showed that a modest reduction in dietary salt intake, in which the use of low-salt bread played a key role, led to a significant, and clinically meaningful, decrease in systolic, but not diastolic, BP in adults with mildly to moderately elevated BP. While acknowledging that reduction of salt in bread involves changes in quality characteristics, e.g., flavour, shelf-life, and texture, as well as in bread manufacturing process [47], the potential impact from a public health perspective in terms of lowering population salt intake justifies further effort by all the relevant stakeholders in relation to striving to achieve the trouble-free production of high-quality, low-salt bread.

## Figures and Tables

**Table 1:** Baseline characteristics of the group of adults with mildly to moderately elevated blood pressure (*n* = 96) who participated in the reduced dietary salt intervention trial.

	Mean (Number)	SD
Age (years)	47.8	9.3
Men:women	(55:41)	-
Height (m)	1.71	0.10
Weight (kg)	81.8	14.6
BMI (kg/m^2^)	27.2	6.4
Systolic blood pressure (mmHg)	138.5	10.4
Diastolic blood pressure (mmHg)	86.5	8.8
Urinary sodium (mmol/day)	108.3	45.7
Current smokers	(11)	-

BMI, body mass index.

**Table 2:** Blood pressure, urinary, and serum biochemical variables in adults with mildly to moderately elevated blood pressure (*n* 96) during the reduced-salt and usual-salt dietary periods.

Dietary Period	Usual-Salt		Reduced-Salt		*p*-Value ^1^
Blood pressure:					
Systolic (mmHg)	134.3	12.1	131.0	11.0	<0.0001
Diastolic (mmHg)	84.7	8.5	84.6	8.2	0.815
Urine ^2^:					
Sodium (mmol/day)	106.0	53.6	77.6	35.6	<0.0001
Potassium (mmol/day)	97.6	51.5	95.9	51.5	0.771
Calcium (mmol/day)	4.36	2.35	4.10	2.52	0.183
NTx (nmol BCE/mmol creatinine)	55.0	36.7	54.6	42.9	0.654
Serum ^3^:					
25(OH)D (nmol/L)	53.6	16.7	52.6	14.7	0.414
BAP (U/L)	27.2	9.3	27.7	9.5	0.137
Osteocalcin (g/L)	12.5	4.3	13.0	5.6	0.208
CTx (g/L)	0.69	0.31	0.69	0.29	0.964
PTH (pg/mL)	64.2	31.0	62.1	30.9	0.271

^1^*P*-value for treatment effect; there was no significant carry-over effects (*p* > 0.13, in all cases). ^2^ Based on *n* = 84 due to exclusion of subjects who missed a urine collection at week 5 or 10 (*n* = 8) or had suspected inaccurate urine collections at week 5 or week 10 (*n* = 4). ^3^ Based on *n* = 90. NTx, N-telopeptides of Type I collagen; BCE, bone collagen equivalents; 25(OH)D, 25-hydroxyvitamin D; BAP, Bone-specific alkaline phosphatase; CTx, C-telopeptides of Type I collagen; PTH, parathyroid hormone.

**Table 3:** Blood lipids in adults with mildly to moderately elevated blood pressure during the reduced-salt and usual-salt dietary periods.

Dietary Period	Usual-Salt		Reduced-Salt		*p*-Value ^1^
Plasma ^2^:					
Total cholesterol (mg/dL)	192.9	33.7	195.9	33.9	0.262
LDL cholesterol (mg/dL)	119.3	29.6	122.0	30.3	0.266
HDL cholesterol (mg/dL)	54.4	14.9	53.9	14.7	0.451
TAG (mg/dL)	96.0	44.9	100.9	46.9	0.184

^1^*P*-value for treatment effect; there was no significant carry-over effects (*p* > 0.67, in all cases). ^2^ Based on *n* = 90. LDL, Low Density Lipoprotein; HDL, High Density Lipoprotein; TAG, Triacylglycerol.

## Supplementary Materials

The following is available online at https://www.mdpi.com/2072-6643/11/8/1725/s1, Table S1: Regression analysis of potential predictors of response of systolic blood pressure.
