# Supplementary material for: ‘Low-Salt’ Bread as an Important Component of a Pragmatic Reduced-Salt Diet for Lowering Blood Pressure in Adults with Elevated Blood Pressure
Source: Nutrients. 2019 Jul 26;11(8):1725. doi: 10.3390/nu11081725 (PMC6722563; doi:10.3390/nu11081725)
Supplement: Supplementary file 1 [file nutrients-11-01725-s001.pdf]

Table S1. Regression analysis of potential predictors of response of systolic blood pressure.

**Predictor**	**B**	**Standard Error**	***P*-value**
Dietary salt period	-6.23	1.81	0.001
Sex	0.75	1.89	0.69
Smoking status	-0.47	2.82	0.87
Vitamin D status	-0.10	0.07	0.19
Age	0.07	0.11	0.51
BMI	-0.20	0.226	0.38

*Additional model including urinary sodium and systolic blood pressure at baseline showed these were non-significant determinants (*P*=0.14 and *P*=0.95, respectively) and dietary salt periods remained a significant predictor (*P*=0.008).
